# Hypothalamic subunit volumes and relations to violence and psychopathy in male offenders with or without a psychotic disorder

**DOI:** 10.1007/s00406-023-01725-4

**Published:** 2024-02-14

**Authors:** Christina Bell, Jaroslav Rokicki, Natalia Tesli, Tiril P. Gurholt, Gabriela Hjell, Thomas Fischer-Vieler, Nina Bang, Ingrid Melle, Ingrid Agartz, Ole A. Andreassen, Petter Andreas Ringen, Kirsten Rasmussen, Hilde Dahl, Christine Friestad, Unn K. Haukvik

**Affiliations:** 1 Department of Psychiatry, Oslo University Hospital, Nydalen, P. O. Box 4956, 0424 Oslo, Norway; 2 Norwegian Centre for Mental Disorders Research (NORMENT), Institute of Clinical Medicine, University of Oslo, Oslo, Norway; 3 Division of Mental Health and Addiction, Norwegian Centre for Mental Disorders Research (NORMENT), Oslo University Hospital, Oslo, Norway; 4 Department of Psychiatry, Østfold Hospital Trust, Graalum, Norway; 5 Division of Mental Health and Addiction, Vestre Viken Hospital Trust, Drammen, Norway; 6 Department of Adult Psychiatry, Institute of Clinical Medicine, University of Oslo, Oslo, Norway; 7 Department of Psychiatric Research, Diakonhjemmet Hospital, Oslo, Norway; 8 Department of Clinical Neuroscience, Centre for Psychiatry Research, Karolinska Institutet & Stockholm Health Care Services, Stockholm Region, Stockholm, Sweden; 9 Centre for Research and Education in Forensic Psychiatry, St. Olavs Hospital, Trondheim, Norway; 10 Department of Psychology, Norwegian University of Science and Technology (NTNU), Trondheim, Norway; 11 Department of Mental Health, Norwegian University of Science and Technology (NTNU), Trondheim, Norway; 12 Centre of Research and Education in Forensic Psychiatry, Oslo University Hospital, Oslo, Norway; 13 University College of Norwegian Correctional Service, Oslo, Norway

**Keywords:** Hypothalamus, Violence, Schizophrenia, Psychopathy, Psychosis, Forensic Psychiatry

## Abstract

The hypothalamus is key to body homeostasis, including regulating cortisol, testosterone, vasopressin, and oxytocin hormones, modulating aggressive behavior. Animal studies have linked the morphology and function of the hypothalamus to aggression and affiliation, with a subregional pattern reflecting the functional division between the hypothalamic nuclei. We explored the relationship between hypothalamic subunit volumes in violent offenders with (PSY-V) and without (NPV) a psychotic disorder, and the association with psychopathy traits. 3T MRI scans (*n* = 628, all male 18–70 years) were obtained from PSY-V, *n* = 38, NPV, *n* = 20, non-violent psychosis patients (PSY-NV), *n* = 134, and healthy controls (HC), *n* = 436. The total hypothalamus volume and its eleven nuclei were delineated into five subunits using Freesurfer v7.3. Psychopathy traits were assessed with Psychopathy Checklist-revised (PCL-R). ANCOVAs and linear regressions were used to analyze associations with subunit volumes. Both groups with a history of violence exhibited smaller anterior–superior subunit volumes than HC (NPV Cohen’s *d* = 0.56, *p* = 0.01 and PSY-V *d* = 0.38, *p* = 0.01). There were no significant differences between HC and PSY-NV. PCL-R scores were positively associated with the inferior tubular subunit on a trend level (uncorrected *p* = 0.045, Cohen’s *d* = 0.04). We found distinct hypothalamic subunit volume reductions in persons with a history of violence independent of concomitant psychotic disorder but not in persons with psychosis alone. The results provide further information about the involvement of the hypothalamus in aggression, which ultimately may lead to the development of targeted treatment for the clinical and societal challenge of aggression and violent behavior.

**Graphical abstract:**

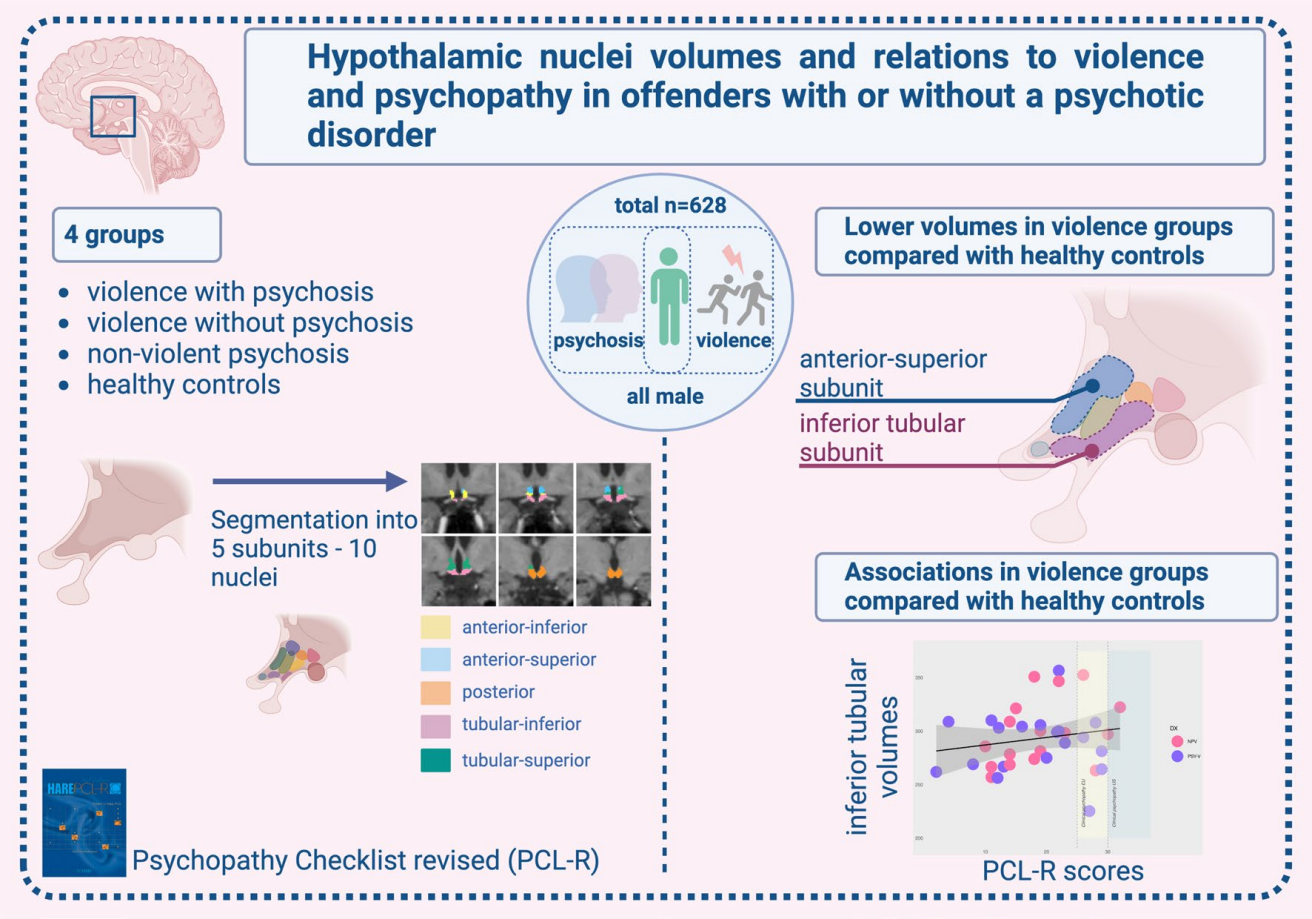

**Supplementary Information:**

The online version contains supplementary material available at 10.1007/s00406-023-01725-4.

## Introduction

Human aggressive behavior is a major health and societal problem, with a higher prevalence among psychiatric patients [20, 23, 27, 45, 65, 68]. Understanding the underlying neurobiological mechanisms for aggression and violence is important to develop targeted treatment and preventive measures [27]. In the forensic setting, violence can be approached as a medical syndrome, deconstructed into three major symptom domains: psychotic, impulsive/reactive, and premeditated [54]. A complicating factor in forensic psychiatry is the comorbidity between psychotic disorders and psychopathic traits [28, 46]. Both schizophrenia and psychopathy are associated with structural and functional brain abnormalities in several key brain systems underlying emotion processing and regulation [54]. They show partly overlapping grey matter loss and aberrant network organization [14, 40], which might reflect the known comorbidity between the two [9, 24, 35, 44].

In schizophrenia patients with a history of violence, the most consistent structural abnormalities are reduced volumes of the hippocampus and the frontal lobe (in particular the orbitofrontal and anterior cingulate cortex) and functional MRI studies have shown aberrant connectivity patterns in the frontal lobe and amygdala [19]. Psychopathy is associated with a reduced volume of the prefrontal cortex and both reduced volume and abnormal shape of the hippocampus and amygdala [14], as well as volumetric abnormalities in the temporal cortex, the cingulate, the insular cortices, and the dorsal and ventral striatum [31]. The striatal deviances are consistent with data from neuropsychological and fMRI studies showing abnormal reward and punishment information processing in individuals with psychopathy [14]. However, the findings differ between studies and no specific biomarkers or brain structural correlates of violence have yet been determined [19].

A region of importance to aggression that to date has received little attention in MRI studies is the hypothalamus, even though this structure is known to be involved in the classic “fight or flight “-response. Surgical experiments performed on cats during the 1920s demonstrated the importance of the hypothalamus in expressing rage [16]. The hypothalamus is part of a broader neural circuit that modulates aggressive behavior and where the amygdala is a key component [27]. For instance, reactive aggression seems to be mediated via a circuit that runs from the medial amygdala downward, largely via the stria terminalis to the medial hypothalamus and from there to the dorsal half of the periaqueductal grey (PAG) [15]. The hypothalamus consists of morphologically distinct nuclei that serve different physiologic functions as the hypothalamus participates in body temperature regulation, appetite and weight, childbirth, growth, breast milk production, sleep–wake cycle, sex drive, emotions, and behavior [49]. Followingly, disorders affecting the hypothalamus can cause different signs and symptoms, depending on the particularly affected nuclei [49].

The stress in the form of childhood adversities (maltreatment) has been intensely researched; as it constitutes a risk factor for both psychosis and future violence [42, 55, 63], often referred to as “the cycle of violence” hypothesis [66]. The hypothalamus is found to be highly relevant for this as psychological stress at various stages during development may produce lasting changes in the hypothalamus–pituitary–adrenal (HPA) axis functioning and thereby increase the risk of later antisocial behavior [61]. Early life stress has persistent and pervasive effects on prefrontal–hypothalamic–amygdala and dopaminergic circuits that are partially mediated by alterations in HPA axis function. This disturbance of brain development happening after stress related to childhood adversities can lead to impairments to the brain circuits involved in cognitive and affective processes, leading to psychopathology, including schizophrenia, antisocial personality disorder, and psychopathy, and hence, increased aggression and violent behavior [10, 56].

Hypothalamic hormonal aberrations involving oxytocin, cortisol, testosterone, and vasopressin (ADH) have been related to aggression [21, 47, 48]. Despite controversies [5], oxytocin is generally thought to promote social affiliative interactions in a wide range of animals, including humans [39, 52]. Cortisol mobilizes the body’s resources and provides energy in times of stress [37]. It is also involved in sensitivity to punishment and withdrawal behavior [50] and potentiating the state of fear [51]. Testosterone is associated with approach-related behavior, reward sensitivity, and fear reduction [25]. Low levels of cortisol, accompanied by high levels of testosterone, might contribute to (primary) psychopathy [62] as demonstrated by showing a significant relationship between psychopathy and the ratio of baseline testosterone to cortisol activity [25]. Vasopressin or antidiuretic hormone (ADH) is released from the posterior pituitary and may predispose individuals to antisocial behavior by interfering with social perception and reducing emotion recognition [60].

Three nuclei of the hypothalamus are of special interest in aggression: the paraventricular nucleus, the preoptic nucleus (both in the anterior–superior subunit), and the dorsomedial nucleus (in the superior tubular subunit). The paraventricular nucleus (PVN) produces corticotropin-releasing hormone (CRH), which regulates ACTH secretion by the pituitary and thereby leads to the secretion of cortisol by the adrenals [49]. The PVN also participates in the production and secretion of oxytocin and vasopressin (ADH). The preoptic nucleus produces and secretes gonadotropin-releasing hormone (GnRH) for sex hormone regulation [49], which ultimately affects the secretion of testosterone from the gonads and the adrenals [25]. The dorsomedial nucleus is an emotional response center [49]. It is a part of the superior tubular subunit, together with PVN and lateral hypothalamus [6]. Electrical stimulation of a circuit including the dorsomedial hypothalamus and the ventral half of the PAG has been shown to initiate predatory attacks in cats and rats [30]. The attack lasts only as long as the stimulus is applied.

Despite the key role of the hypothalamus in aggression, a few studies address the human hypothalamic structure in vivo. As far as we know, no other studies have investigated the hypothalamus in severe mental disorders and violence. Here, we will use a recently developed automated segmentation of the hypothalamus and its subunits from the Freesurfer 7.3 software tool [8] to investigate the association between volumes of hypothalamic subunits and violence and psychosis. Generally, smaller grey matter volumes in the brain indicate a lack of neurons and are associated with a reduction in daily function [41]. We hypothesized (1) that subunits involved in the release or production of cortisol, testosterone, oxytocin, and vasopressin would differ in the violent groups (violent offenders with a psychotic disorder (PSY-V) and non-psychotic violent (incarcerated) offenders (NPV), that is mainly the anterior–superior subunit which includes the PVN and the preoptic area (2) that there would be a difference in the superior tubular subunit, which includes the dorsomedial nucleus (as well as PVN) because of previous studies suggesting that this unit is related to aggression (since electrical stimulation of this region has been shown to lead to aggressive behavior) [30] (3) that higher levels of psychopathic traits would be associated with differences in the volumes of these two subunits (that is the anterior–superior subunit and the superior tubular subunit) (4) that total hypothalamic volume would be smaller in the groups with psychotic disorders, the PSY-V-group, and the psychosis non-violent group (PSY-NV). Accordingly, the PSY-V group would have two reasons for having smaller volumes, (i.e., 1. psychotic disorder and 2. violence). Hence, we hypothesize that this group generally would have the lowest volumes (both total volume and nuclei volumes).

## Materials and methods

### Sample

The subject sample (*n* = 628) consisted of four groups of male participants, and their diagnoses were based on the Diagnostic and Statistical Manual of Mental Disorders (DSM-IV).

The violent offenders with psychotic disorders (PSY-V) group (*n* = 38) consisted of patients predominantly within the schizophrenia (SCZ) spectrum (i.e., schizophrenia, schizoaffective disorder, and schizophreniform disorder). In addition to diagnosis, the inclusion criteria were a history of murder or attempted murder or severe physical assault towards other people (including sexual assaults). The PSY-V group was recruited from high-security psychiatric wards at Oslo University Hospital and Østfold Hospital, Norway.

The violent offenders without a psychotic disorder (NPV) group (*n* = 20) consisted of prisoners serving a preventive detention sentence in Norway, due to the perpetration of a crime involving serious interpersonal violence, also defined as a history of murder or attempted murder or severe physical assault towards other people (including sexual assaults). They did not have a psychotic disorder at the time of the violent offense nor at study inclusion.

The non-violent patient group with a psychotic disorder (PSY-NV) group (*n* = 134) consisted of individuals with a schizophrenia spectrum disorder without a history of violence. The PSY-NV were recruited from four major psychiatric hospitals and affiliated outpatient clinics from the Oslo region, Norway.

The non-violent, non-psychotic healthy control (HC) group (*n* = 436) consisted of persons with no history of severe mental disorder and was randomly selected from the Norwegian national population registry (https://www.ssb.no/en). All were residents of the Oslo region, Norway, and they were invited by a personal letter to participate in the study.

All participants were included as part of the ongoing multi-centre TOP (Thematically organized psychosis) or the “Violence in severe mental disorder” (sTOP) study in Oslo, Norway, between 2015 and 2019. Inclusion criteria for all groups were age between 18 and 70 years, Norwegian language knowledge to understand the study protocol and procedures, IQ scores above 65, and the ability to give informed consent to study participation. Additional inclusion criteria for the PSY-V and NPV participants were safety evaluations regarding study procedures and permission to leave the hospital ward/prison for the MRI acquisition. Exclusion criteria for all groups were head trauma leading to loss of consciousness for more than 10 min and somatic illness that might have affected brain morphology. The subject sample is overlapping with previous studies assessing amygdala volumes [2], white matter microstructure [58], age deviations in psychosis and psychopathy [57], and psychopathy traits [3].

The study was approved by the Norwegian Regional Committee for Medical Research Ethics, Norwegian Data Inspectorates, and relevant correctional agencies. Written informed consent was obtained from all participants after a complete description of the study and after the project physicians or the treating psychiatrist/psychologist had evaluated the subject’s capacity to give informed consent to study participation. The study was conducted according to the Helsinki declaration.

### Clinical assessment

Trained physicians, psychiatrists, and psychologists administered assessments of each study participant through clinical examination, including blood samples for clinical-chemical analyses to detect somatic illness.

The patient's psychiatric diagnoses were confirmed with the Structured Clinical Interview for DSM-IV axis 1 disorders (SCID-1) and supplementary information drawn from medical records. The PSY-V group had diagnostic evaluations based on detailed medical records and forensic reports. Psychosocial functioning was evaluated with the Global Assessment of Functioning scale (GAF) scale. Alcohol and illicit substance use were evaluated with The Alcohol Use Disorder Identification Test (AUDIT) and The Drug Use Disorder Identification Test (DUDIT). Current psychotic symptoms were rated using the Positive and Negative Syndrome Scale (PANSS). Medication use was assessed, and Defined Daily Dosages (DDD) of current antipsychotic medication use were calculated per the guidelines from the World Health Organization (https://www.whocc/atc_ddd_index/).

Among PSY-V and NPV, a history of violence was assessed based on court documents and hospital records. Psychopathy traits were assessed with the Hare Psychopathy Checklist-Revised (PCL-R) based on interviews, court documents, and/or medical records. The PCL-R is a 20-item scale for assessing psychopathy in research, clinical, and forensic settings. It uses a semi-structured interview, file, and collateral information to measure personality traits and behaviors related to a widely understood conception of psychopathy.

To confirm the absence of a previous history of violence in the PSY-NV group, the medical records were carefully inspected. This procedure encompassed the evaluation of all study inclusion protocols which are based on comprehensive information obtained from medical records, including data from clinical journals and detailed interviews with the patient.

HC subjects were screened with the Primary Care Evaluation of Mental Disorders (Prime-MD) questionnaire and interviewed by specially trained clinical psychologists or neuroscientists to confirm no history of severe psychiatric disorder.

Current IQ was measured in all participants with the Norwegian version of the Wechsler Abbreviated Scale of Intelligence (WASI) by specially trained psychologists. For each participant, the number of completed years of formal schooling was used as an estimate for years of education.

### MRI acquisition and post processing

MRI data were acquired using two GE 3T scanners due to a hardware upgrade. The MRI data obtained before the upgrade were collected on a 3T GE Signa HDxt scanner (GE Medical Systems, Milwaukee, WI, USA) using a standard eight-channel head coil at Oslo University Hospital, Norway. T1-weighted volumes were acquired using a sagittal 3D fast spoiled gradient echo (FSPGR) sequence with the following parameters: repetition time (TR) 7.8 ms, echo time (TE) 2.9 ms, flip angle 12°, slice thickness 1.2 mm, 166 slices, field of view (FOV) 256 mm × 256 mm, acquisition matrix 256 × 192 mm, reconstructed in-plane resolution 256 × 256 mm/pix. MRI data after the upgrade were collected on a 3T GE 750 Discovery scanner using a 32-channel head coil at Oslo University Hospital. T1-weighted volumes were acquired using a sagittal 3D BRAVO sequence with the following parameters: repetition time (TR) 8.2 ms, echo time (TE) 3.2 ms, flip angle 12°, slice thickness 1.0 mm, 192 slices, and field of view (FOV) 256 mm × 256 mm. A neuroradiologist evaluated all MRI scans to ensure no brain pathology affecting the analyses.

T1-weighted MRI volumes were pre-processed using the standard FreeSurfer recon-all pipeline (version 7.1; http://surfer.nmr.mgh.harvard.edu/). An automated segmentation of hypothalamic subunits was performed using FreeSurfer v7.3 based toolbox (Billot B, 2022). Anatomically, the hypothalamus can be organized in different ways, and the current segmentation tool (Freesurfer 7.2) separates five subunits comprising 11 nuclei, as follows: 1. Anterior–inferior subunit (suprachiasmatic nucleus, supraoptic nucleus (SON)), 2. Anterior–superior subunit [preoptic area, paraventricular nucleus (PVN)], 3. Posterior subunit (mammillary body, lateral hypothalamus, tuberomammillary nucleus (TMN), 4. Inferior tubular subunit (arcuate nucleus, ventromedial nucleus, SON, lateral tubular nucleus and TMN), and 5. Superior tubular subunit (dorsomedial nucleus, PVN, lateral hypothalamus) [8].

### Statistical analyses

We used R software (4.1.2) for statistical analyses.

For the descriptive statistical analyses, we used analyses of variance (ANOVA) with post hoc Tukey tests or *t* tests to assess group differences in demographic and clinical characteristics. All statistical tests were two-tailed, with statistical significance reported at 0.05.

To assess group differences, first, we performed ANCOVA controlling for age, ICV, and substance use (AUDIT and DUDIT), adjusting for the number of subunits (five subunits and total hypothalamus volume) using a conservative Bonferroni correction. Right and left hemisphere hypothalamic subunit volumes were merged as we did not have any laterality hypotheses and to reduce the number of tests. Subunits with significant group differences were further investigated with pairwise comparisons using general linear models (GLM) as implemented in the permutation analysis of linear models (PALM) [67] toolbox with 10,000 permutations while controlling for effects of age and ICV, and demeaning the data including covariates in the design matrix. We adjusted the resulting p values for the number of groups using Bonferroni correction. Effect sizes were calculated with Cohen's *d*.

In the next step, we explored associations between psychopathy traits (PCL-R scores) and hypothalamic total and subunit volumes in the two groups with a history of violence (PSY-V and NPV) by using a GLM in the PALM toolbox with 10,000 permutations, with PCL-R as the independent variable, additionally controlling for the effects of age, ICV, and psychotic disorder status in a separate model. Bonferroni correction was used to adjust for the number of subunits.

## Results

### Clinical and demographic characteristics

Clinical and demographic statistics are summarized in Table 1. There were significant differences between groups in age, illicit substance use, total years of education, DDD, IQ, and GAF function, with higher age in the violent offender group without psychotic disorders (NPV) and PSY-V having the highest substance abuse and lowest education, highest doses of antipsychotic medication, and lowest IQ and GAF function. All three PANSS subscores and CDSS also differed between groups. Significant differences were found on the scanner (TOP3GE vs. TOP 3T) (*F* = 0.0082, *p* = 0.008).

**Table 1:** Clinical and demographic characteristics

	HC (*n* = 436)	NPV (*N* = 20)	PSY-NV (*N* = 134)	PSY-V (*N* = 38)	Total (*N* = 628)	*F* value	*P* value
Age (years), Mean and SD	35 ± 9	42 ± 14	29 ± 9	35 ± 9	34 ± 9	21	< 0.001
IQ WASI, Mean and SD (*n* = 389/17/124/19)	115 ± 10	102 ± 13	102 ± 13	94 ± 15	111 ± 13	57	< 0.001
Education, Mean and SD (*n* = 385/0/124/21)	14.6 ± 2.3		12,3 ± 1.9	12.1 ± 2.6	14.0 ± 2.4	62	< 0.001
PANSS total, Mean and SD (*n* = 0/20/133/34)		39 ± 11	61 ± 17	65 ± 19	59 ± 18	16	< 0.001
PANSS general, Mean and SD (*n* = 0/20/133/34)		22 ± 7	31 ± 9	31 ± 9	30 ± 9	11	< 0.001
PANSS negative, Mean and SD (n = 0/20/133/35)		9 ± 2	16 ± 6	18 ± 7	14 ± 6	15	< 0.001
PANSS positive, Mean and SD (*n* = 0/20/133/34)		9 ± 4	14 ± 5	16 ± 8	14 ± 6	12	< 0.001
GAF-S, Mean and SD (*n* = 0/0/132/34)			48 ± 14	42 ± 11	47 ± 13	7	0.009
GAF-F, Mean and SD (*n* = 0/0/130/34)			48 ± 13	39 ± 8	46 ± 13	15	< 0.001
CDSS, Mean and SD (n = 0/0/127/33)			4.5 ± 4.3	1.7 ± 2.1	3.9 ± 4.1	13	< 0.001
AUDIT, Mean and SD (*n* = 336/11/120/30)	5.77 (3.22)	8.55(12.3)	6.49 (6.34)	4.73 (4.65)	5.94 (4.61)	2.6	0.051
DUDIT, Mean and SD (*n* = 336/11/120/30)	0.39 (1.62)	6.18(11.8)	5.75 (8.97)	9.47 (10.1)	2.36 (6.21)	48	< 0.001
DDD, Mean and SD (*n* = 110/33)			1.17(0.807)	1.62(0.78)	1.28(0.814)	8.2	0.005
PCL-R, Mean and SD (*n* = 20/26)		20.7(7.7)		19.3 (9.1)	20 (8.4)		p = 0.61
Scanner							
TOP3GE	262 (60.1%)	20 (100%)	81 (60.4%)	26 (68.4%)	389 (61.9%)	14	0.003
TOP3T	174 (39.9%)	0 (0%)	53 (39.6%)	12 (31.6%)	239 (38.1%)		

*HC* healthy controls, *NPV* Non-psychotic violent offenders, *PSY-V* violent offenders with psychosis, *PSY-NV* non-violent patients with psychosis, *SD* standard deviation, *GAF* Global Assessment of Function scale split version, *PANSS* Positive and Negative Syndrome Scale, *AUDIT/DUDIT* Alcohol Disorders Identification Test/Drug Use Disorder Identification Test, *CDSS* Calgary depression scale, *DDD* defined daily dose, *PCL-R* Psychopathy Checklist-Revised

There were no significant differences on a group level for alcohol abuse or GAF symptoms and no significant differences between PSY-V and NPV in total psychopathy scores. The latter was already known from an earlier study on psychopathy subdomains [4].

### Hypothalamus and subunit volumes

The ANCOVAs showed that the anterior superior subunit differed significantly from the others (*F* = 4.73, *p* = 0.003, corrected *p* value 0.017). None of the other subunits differed between groups. Further pairwise comparisons using general linear models (GLM) revealed that both groups with a history of violence exhibited smaller anterior superior subunit (preoptic area and paraventricular nucleus) volumes than HC (NPV Cohen’s *d* = 0.56, *p* = 0.01 and PSY-V *d* = 0.38, *p* = 0.01). There were no significant differences between HC and PSY-NV. Correcting for alcohol- and drug use with AUDIT and DUDIT did not influence the results (the anterior superior subunit still differed significantly from the others; *F* = 4.3, *p* = 0.005, see Table 4 under supplemental information).

See Tables 2 and 3 for the results regarding hypothalamic subunit volumes.

**Table 2:** Group differences in hypothalamic subunit volumes between HC, NPV, PSY-V, and PSY-NV

	*F* value	*P *value (uncorrected)	*P *value (corrected)
Anterior-inferior subunit	2.26	0.08	0.48
Anterior–superior subunit	4.73	**0.003**	**0.017**
Posterior subunit	0.40	0.755	1.000
Tubular-inferior subunit	0.92	0.43	1.000
Tubular-superior subunit	0.93	0.43	1.000
Whole hypothalamus	0.72	0.54	1.000

*p* value (corrected): corrected with Bonferroni for the number of subunits

The alpha-level for significance was set to 0.05 (in bold)

**Table 3:** Significant group differences (in the anterior–superior subunit) were further investigated with pairwise comparisons using general linear models (GLM)

Contrast	Cohen’s *d*	*p*-uncorrected	*p*-corrected
HC > NPV	0.56	**0.010**	**0.038**
HC < NPV	– 0.56	0.991	1.000
HC > PSY-NV	– 0.02	0.568	1.000
HC < PSY-NV	0.02	0.432	1.000
HC > PSY-V	0.38	**0.012**	**0.047**
HC < PSY-V	– 0.38	0.988	1.000
NPV > PSY-V	– 0.18	0.739	1.000
NPV < PSY-V	0.18	0.261	1.000
PSY-NV > PSY-V	0.40	**0.017**	0.069
PSY-NV < PSY-V	– 0.40	0.983	1.000

Results are Bonferroni-corrected for the number of groups (4)

The alpha-level for significance was set to 0.05 (in bold)

### Psychopathy traits and subunit volumes

Psychopathy scores correlated positively with the volume of the inferior tubular subunit on a trend level, even when we controlled for psychotic disorder, however, this result did not remain significant after adjustment for multiple comparisons (uncorrected *p* = 0.045 Cohen’s *d* = 0.04).

**Fig. 1:**
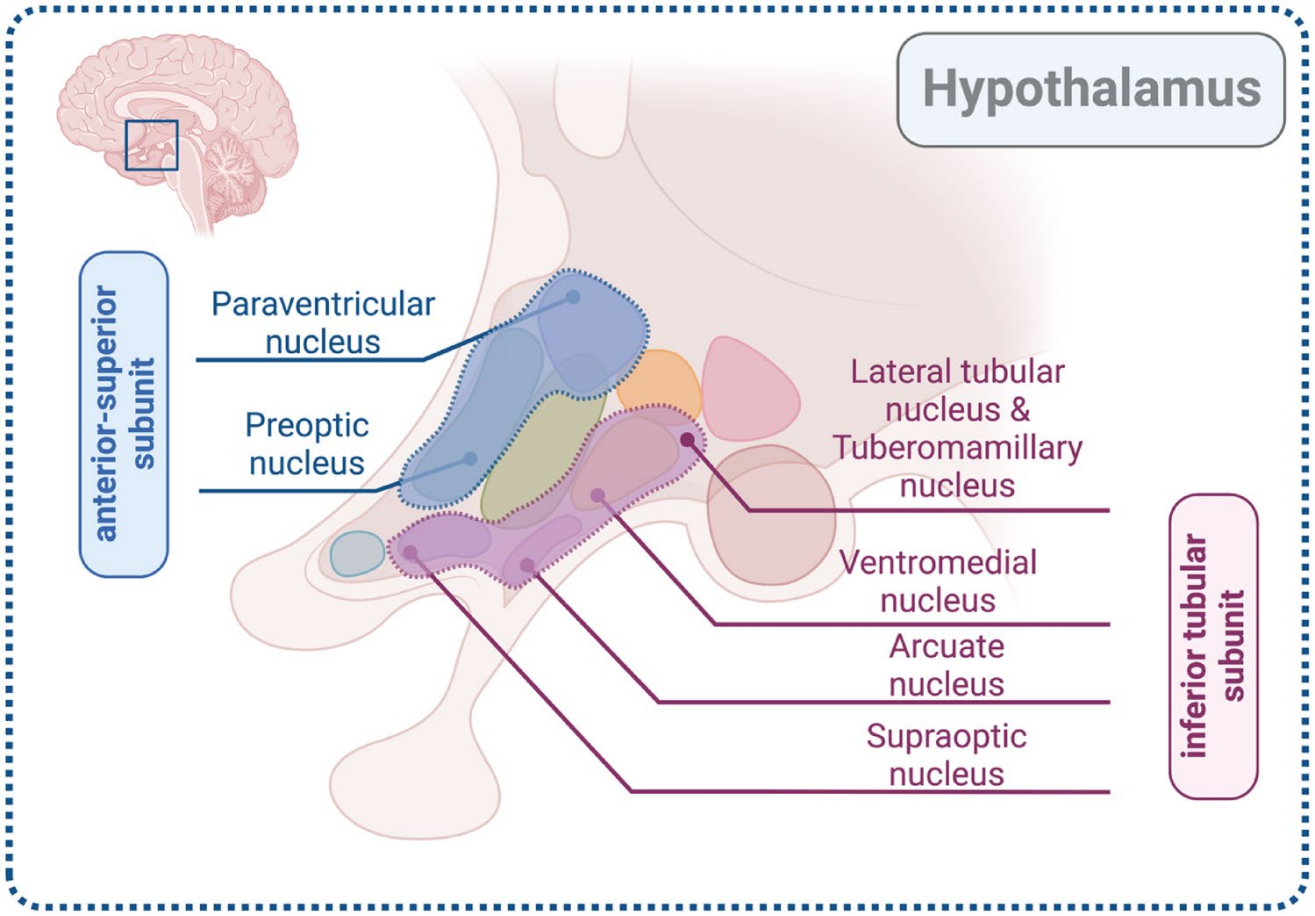
Subdivisions of the hypothalamus, focusing on the anterior–superior subunit and the inferior tubular subunit (Biorender.com)

See Fig. 1 for subdivisions of the hypothalamus.

**Fig. 2:**
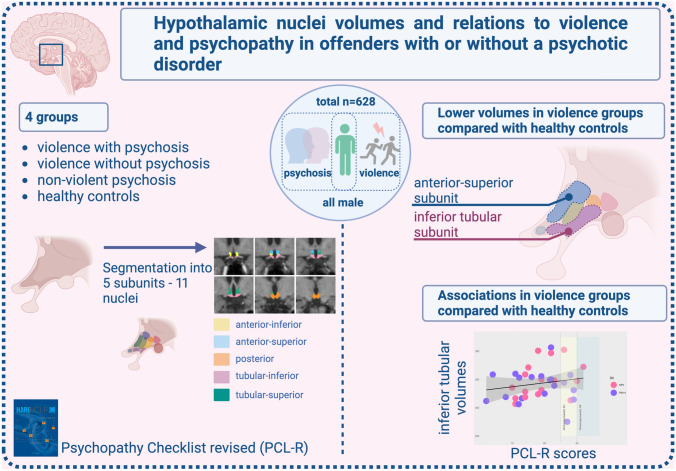
The volume of the anterior–superior subunit of the hypothalamus was smaller in the groups with a history of violence (NPV and PSY-V) than in healthy controls (HC)

See Fig. 2 for the results regarding the hypothalamic subunits.

## Discussion

In this study, we explored hypothalamic subunits in relation to violence, psychotic disorders, and psychopathy. We found that violent offenders with and without a psychotic disorder exhibited reduced anterior superior subunit (which comprises the paraventricular nucleus and the preoptic area) compared to HC. Still, we found no significant differences between HC and PSY-NV. Further, we found psychopathy traits *positively* associated with the *inferior* tubular subunit on a trend level.

Cumulative scientific evidence has shown that aggressive behavior is linked to abnormal hormonal levels [13, 21, 53], particularly oxytocin, cortisol, vasopressin, and testosterone. Interestingly, the subunit that differed between the groups with or without a history of violence is the subunit whose nuclei are most involved in the production or secretion of these aggression-related hormones. The anterior superior subunit includes two nuclei: the paraventricular nucleus (PVN) and the preoptic nucleus. The PVN is involved in the production and secretion of several hormones, predominantly oxytocin, vasopressin, and cortisol. Further, it contains neurons that secrete glutamate and angiotensin II, which induce sympatho-excitatory effects. The preoptic nucleus produces and secretes the gonadotropin-releasing hormone (GnRH) for sex hormone regulation [49].

A key aspect of aggression regulation is the hormonal response system activated by mental and physical stressors [32], including the HPA axis where cortisol is the end product. Cortisol is involved in fear and stress responses, as well as aggression, and low levels of cortisol have been linked to psychopathy [47]. Aggressive individuals have been suggested to exhibit a negative coupling between HPA and HPG with low cortisol and high testosterone levels, as well as low oxytocin levels, but the findings are inconsistent [21]. This so-called high T/C ratio leads to low sensitivity to stress, increased attention to threatening cues, decreased emotional recognition, and salience of dominance and approach motivation [21]. Low levels of cortisol and high testosterone can make you less afraid [11, 29]—which is in line with the low fear theory of primary psychopathy [64].

Low oxytocin levels may reduce empathy [1], and oxytocin plays a significant role in stress response and is considered to have an anxiolytic effect, partly because it is released directly to decrease cortisol [43]. Oxytocin has been widely investigated for its role in social-affective behaviors, including social affiliation, emotional recognition, pair bonding, trust, empathy, and attachment, all of which may be impaired in aggressive individuals [21]. There is some evidence, suggesting that the administration of testosterone leads to decreased connectivity of the orbitofrontal cortex (OFC) with the amygdala, whereas oxytocin administration enhances the connectivity between these two regions [12]. As mentioned, vasopressin seems to predispose individuals to antisocial behavior by disturbing social perception [60], and this hormone is also found to induce a more longstanding fear response in the amygdala after threatening stimuli [69]. There is compelling evidence from several mammalian species including humans that vasopressin enhances aggression, and vasopressin antagonists block aggressive behavior in rats [18]. The vasopressin activity appears linked to the serotonin system providing a mechanism for enhancing and suppressing aggressive behavior [17].

Studies have shown that brain function parallels changes in brain structure [33] and that, generally, lower volumes of grey matter can often lead to dysfunction [26]. We may only speculate that there is a dysfunction or a non-optimal function in the anterior superior subunit in our groups with a history of violence, since this was found to have a smaller volume. *This could involve hormone disturbances, maybe in the direction of high testosterone and/or vasopressin and/or low cortisol and/or oxytocin.* Especially, a high T/C ratio could potentially lead these individuals to be more violence prone. Further, our results support the possibility that aberrancies in the production of glutamate and angiotensin II, both of which induce sympatho-excitatory effects, may lead to either higher impulsivity or a loss of inhibition, and thereby affect these groups towards more aggressive behavior.

The volume of the inferior tubular subunit, which was *positively* associated with psychopathy on a trend level, contains the following four nuclei: The arcuate nucleus, the ventromedial nucleus, the supraoptic nucleus (SON), and the lateral tubular nucleus (TMN). The arcuate nucleus releases growth hormone-releasing hormone (GHRH) and dopamine, and the ventromedial nucleus is the center of satiety or fullness. SON resembles the paraventricular nucleus, but its primary function is vasopressin secretion. The lateral tubular nucleus (TMN) has receptors for CRH, somatostatin, N-methyl-D-aspartate (NMDA), and benzodiazepines, as well as muscarinic and cholinergic receptors, and this nucleus is affected in several neurodegenerative diseases, such as Parkinson's, Alzheimer’s, and Huntington’s disease [36]. Maybe, a higher release of dopamine in the arcuate nucleus and/or an increased production of vasopressin in the SON could lead to more aggression as seen in psychopathy.

Interestingly, in support of the hypothalamus being relevant for aggression is this recent GWAS of antisocial personality disorder, where they found the most significant results in a SNP associated with the FOXP2 gene. This gene seems mostly expressed (when it concerns the brain) in the hypothalamus, and 2nd strongest in the frontal cortex [59].

We did not find a difference in the superior tubular subunit, and we did not find higher levels of psychopathic traits to correlate negatively with the volumes of any nuclei. Neither did we find any differences regarding total hypothalamic volume. A recent study [22] investigated structural alterations of the hypothalamus in relation to catatonia. Fritze et al. used the same segmentation algorithm as we did and found that patients with schizophrenia spectrum disorder (SSD) and catatonia had a significantly smaller anterior inferior subunit in the hypothalamus compared to SSD patients without catatonia. Catatonia is related to anxiety and sensitivity to stress [34], and it is a clinical phenomenon that differs from aggression and violence in many ways. Hence, even though these results need replication, it is interesting that the volume reductions associated with catatonia affect other hypothalamic nuclei than the nuclei associated with violence in the current study.

The study's findings must be interpreted cautiously as limitations must be considered. The most significant is the small sample size, particularly for the NPV group. Although the sample included around 20% of individuals serving a preventive detention sentence in Norway at the time of data collection, the results may not be generalizable to a larger population. The study included males only. The sexes are different regarding hormonal functions and aggressive behavior; hence, the findings do not necessarily generalize to women. We only had AUDIT and DUDIT scores for drug screening measuring intake/problematic use during the last 12 months. The AUDIT and DUDIT scores were scored at the time of the MRI scan. Unfortunately, we did not have the scores at the time of the crime. Both the incarcerated group and the hospitalized patient group had few opportunities to use drugs or drink alcohol in the previous year before participating in the study. Still, some patients /incarcerated individuals have used drugs or alcohol in hospital /prison or on leaves from their institution. The tests do not convey information about earlier use.

We did not differentiate the violence according to whether it was psychotic, premeditated, or impulsive, which could have given additional information. We have previously shown an association between childhood adversities and violence in schizophrenia [55], but did not explore possible associations between childhood trauma and hypothalamus subunit volumes. Additionally, not having relevant hormone measurements (blood/urine/saliva) may have limited the ability to determine the hormonal associations of the brain anatomical findings.

Despite these limitations, the study has several strengths. First, all participants underwent a thorough and standardized clinical characterization using validated scoring instruments. Second, examining hypothalamic nuclei can be challenging due to their small size, unclear boundaries, and anatomical variability. [38] However, the novel segmentation algorithm provided by Freesurfer v7.3 is suitable and well-validated [7]. All PCL-R-raters (CB, CF, GH, TFV, and UKH) underwent the same structured training, and raters discussed their scores to achieve consensus. Two raters (CB, UKH) scored both patients with psychotic disorders and violent offenders without psychotic disorders to ensure the same practice in the prisons and hospital wards.

## Conclusion

Here, we found distinct hypothalamic subunit volume reductions in individuals with a history of violence independent of concomitant psychotic disorder but not in individuals with a psychotic disorder alone. Hence, the observed difference may be related to the violent behavior. The subunit involved contains nuclei participating in the production of hormones that are known to affect aggression and violence. The results, if replicated, provide further information about the involvement of the hypothalamus in human aggressive behavior. This may ultimately lead to the development of targeted treatment for the clinical and societal challenge of aggression and violent behavior.

## Supplementary Information

Below is the link to the electronic supplementary material.

Supplementary file1 (DOCX 16 kb)

## Data Availability

This study used the *Services for Sensitive Data* (TSD), University of Oslo, Norway, with resources provided by UNINETT Sigma2—the National Infrastructure for High-Performance Computing and Data Storage in Norway. Due to ethical and data security issues related to the sensitive nature of the clinical data, we are not allowed to share the data without specific IRB approval and data use agreements with the relevant institution. More information can be obtained through the corresponding author, Christina Bell (email address: chrbell@ymail.com).
