# Supplementary material for: Hypothalamic subunit volumes and relations to violence and psychopathy in male offenders with or without a psychotic disorder
Source: Eur Arch Psychiatry Clin Neurosci. 2024 Feb 14;276(3):1301–12. doi: 10.1007/s00406-023-01725-4 (PMC13002772; doi:10.1007/s00406-023-01725-4)
Supplement: Supplementary file 1 — Supplementary file1 (DOCX 16 kb) [file 406_2023_1725_MOESM1_ESM.docx]

**Supplementary information for:**

# Hypothalamic subunit volumes and relations to violence and psychopathy in male offenders with or without a psychotic disorder

*Authors:* Christina Bell^1,2^, Jaroslav Rokicki^3,12^, NataliaTesli^2,3^, Tiril P Gurholt^2,3^, Gabriela Hjell^2,4^, Thomas Fischer-Vieler^2,5^, Nina Bang^11^, Ingrid Melle^2,3^, Ingrid Agartz^2,7,8^, Ole A Andreassen^2,3^, Petter Andreas Ringen^1,2,6^, Kirsten Rasmussen^9, 10,^ Hilde Dahl^9^, Christine Friestad^11,12^, Unn K Haukvik^2,6,11^

**Corresponding author: Christina Bell, email: chrbell@ymail.com, postal address: Oslo University Hospital, P.O.Box 4956 Nydalen, 0424 Oslo, Norway.* Christina Bell: ORCID: <https://orcid.org/0000-0001-8061-1626>

|  | **F-value** | **p-value (uncorrected)** | **p-value (corrected)** |
| --- | --- | --- | --- |
| **Anterior-inferior subunit** | 2.49 | 0.059 | 0.36 |
| **Anterior-superior subunit** | 4.30 | 0.005 | 0.031 |
| **Posterior subunit** | 1.16 | 0.326 | 1.000 |
| **Tubular-inferior subunit** | 1.78 | 0.149 | 0.90 |
| **Tubular-superior subunit** | 0.79 | 0.50 | 1.000 |
| **Whole hypothalamus** | 0.52 | 0.67 | 1.000 |

**Table 4**. Group differences in hypothalamic subunit volumes between HC, NPV, PSY-V and PSY-NV when controlling for substance use (with AUDIT and DUDIT). P-value (corrected): corrected with Bonferroni for the number of subunits.
